# Fatal, self-inflicted injury caused by a pepper spray gun

**DOI:** 10.1007/s00414-025-03645-5

**Published:** 2025-11-19

**Authors:** Zsofia Hajdu, Brita Leyrer, Clemens Reiter

**Affiliations:** 1 Department of Forensic Medicine, Center for Forensic Science, Medical University of Vienna, Vienna, Austria; 2 Armaments and Defence Technology Agency (ARWT), Felixdorf Test and Firing Range, Federal Ministry of Defence, Felixdorf, Austria

## Abstract

Pepper spray guns have gained popularity as self-defense weapons in recent years. Marketed as “less-lethal,” these devices are designed to be easy to use, accurate, and capable of deterring assailants without causing permanent harm. We present a case of a 53-year-old man who sustained a fatal injury caused by a JPX6 Jet Protector pepper spray gun. The device was found in close proximity to the body at the scene. External examination revealed a small entry wound and a triangular, burned stretch zone in the right inguinal area. The cause of death was exsanguination due to laceration of the right internal iliac artery (A. iliaca interna dextra). To our knowledge, this is the first documented fatality caused by a JPX6 Jet Protector.

Following this incident, we have conducted a series of ballistic soap experiments using the JPX6 to evaluate its potential for skin and soft tissue penetration, especially at distances shorter than the manufacturer’s recommended safety distance of 1.5 meters. Our findings confirmed tissue-penetrating capacity at distances as short as point-blank, 10 cm, 20 cm, and 40 cm.

We emphasize the potentially lethal capabilities of pepper spray launchers, particularly when used at close range and by untrained individuals, and advocate their recognition as potential weapons of homicide during forensic scene investigation.

## Introduction

Pepper spray was initially invented during World War I and has since become a widespread tool for self-defense and law enforcement applications [1]. In the United States, it was first used operationally by the FBI in 1973 due to its ability to temporarily incapacitate individuals without inflicting permanent harm. It is also commonly used to deter wildlife [2]. Modern pepper sprays contain oleoresin capsicum (OC), which produces immediate irritation of the skin, eyes, and respiratory system [2]. However, traditional canister sprays are limited in their range, susceptible to blowback in windy conditions and often lack accuracy – particularly in high-stress situations [3].

These limitations led to the development of pepper spray launchers - firearm-like devices offering greater precision and range. The JPX series by Piexon uses a patented jet delivery system to fire a liquid irritant (Piexol 400 kS) at speeds up to 80,56 m/s. Piexol is a highly concentrated cayenne pepper extract with a Scoville value of 400,000. Exposure causes temporary visual impairment, mucosal irritation, coughing and nausea. The incapacitating effects last up to 45 min and typically resolve without long-term consequences [4].

Although pepper spray devices are classified as less-lethal, they are not entirely devoid of risk. According to NATO guidelines, less-lethal weapons are intended to minimize - but not eliminate - the risk of permanent injury or death [5]. The first skin-penetrating injury caused by a pepper spray launcher was reported in 2022 [6], and a fatality involving a JPX4 device was published in the same year [7].

## Case report

We report the case of a 53-year-old man who died from exsanguination following a gunshot-like injury caused by a JPX6 Jet Protector pepper spray launcher. The liquid jet struck the right inguinal region, lacerating the right internal iliac artery (A. iliaca interna dextra), which led to a massive hemorrhage. Toxicological analysis revealed significant alcohol intoxication (2.5‰ in blood and 2.4‰ in urine) at the time of the event. The injury was most likely accidental and self-inflicted at close range.

**Fig. 1:**
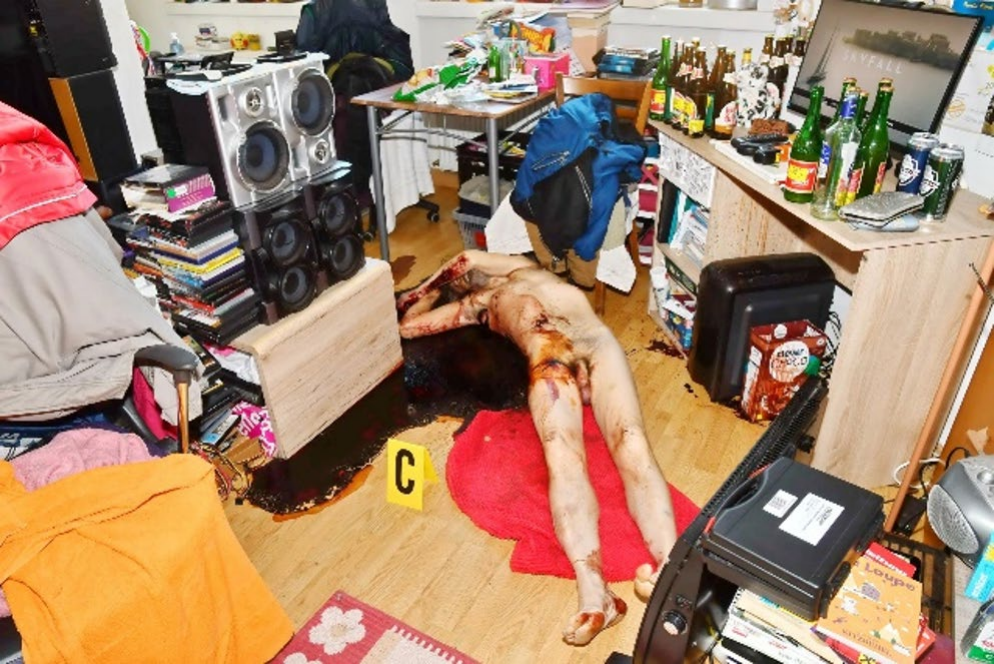
General overview of the discovery site, showing the original position of the body and surrounding environment

**Fig. 2:**
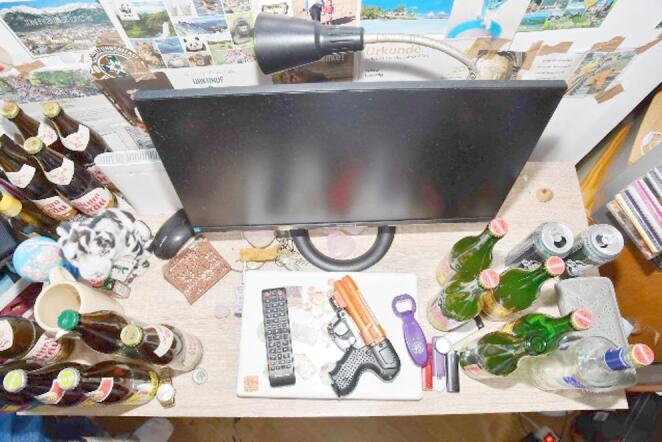
Overview of the table at the scene; the JPX6 device is visible among other items at its final position

Initially, the pepper spray gun was not suspected as the cause of the fatal injury. Forensic medical examination was requested due to dried blood in the victim’s axillary region, raising suspicion of a stab wound. At the scene, the apartment was found in disarray with numerous full and empty bottles of alcoholic beverages. The deceased was lying naked, supine in a pool of blood. Dried blood covered the right side of the chest, with multiple runoff patterns directed posteriorly. The underlying skin had an orange discoloration. An autopsy was ordered and initiated immediately after crime scene documentation (Fig. 1 and 2).

**Fig. 3:**
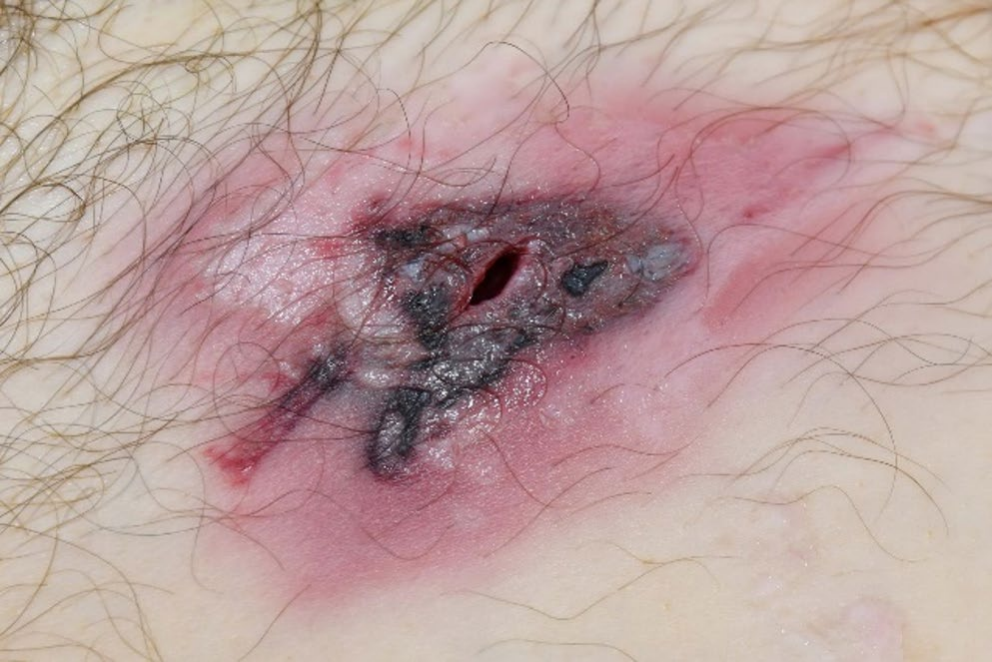
Close-up of the entry wound in the right inguinal region

No reliable medical history was available except for anecdotal information from neighbors stating the man had undergone inguinal hernia surgery weeks or months earlier and complained of persistent postoperative pain, but had not sought further treatment. The decedent had a BMI of 20.6, was 179 cm tall, and weighed 66 kg. While cleaning the body, the orange discoloration in the right lower abdomen, thigh, and inguinal area - initially presumed to be surgical disinfectant - could be easily wiped off. Three small, hypopigmented scars (1.4–2.2 cm) were observed in the abdominal region, interpreted as residuals from laparoscopic abdominal surgery. The right inguinal area was swollen and showed a ~ 0.8 cm long, slit-like, smooth-edged wound, interpreted as an entry wound. This was surrounded by a triangular stretch zone (3.2 × 1.8 cm) with burn markings. Above this zone was an angular lesion (1.5 × 0.3 cm), resembling a partial muzzle imprint. The surrounding skin showed multiple small, round, epidermal abrasions (Fig. 3).

Dissection revealed laceration of both, the anterior as well as the posterior wall of the right internal iliac artery. No projectile or exit wound was present. Deep tissues showed massive hematoma. Signs of severe blood loss were evident, including subendocardial hemorrhages in the heart and a flaccid spleen. The abdominal wall revealed adhesions and a surgical mesh, with no signs of inflammation. Histology confirmed acute hemorrhage in vessel walls and surrounding tissue, with no evidence of inflammation.

Toxicological screening of blood, urine, and brain tissue revealed traces of Paliperidone, an atypical antipsychotic (active agent in Xeplion^®^), used to treat schizophrenia. Medical records confirmed inguinal hernia surgery approximately five months earlier.

## Functional principle of the JPX6 irritant delivery device

The JPX6 is a reusable, less-lethal irritant delivery system designed for law enforcement, security personnel, and civilian self-defense applications. The device consists of a polymer-based grip unit with a mechanical double-action trigger and an interchangeable magazine capable of delivering four individual shots. Each shot is contained in an aluminum cartridge embedded within a plastic magazine that includes integrated nozzles, ignition units, and propellant cartridges.

The firing mechanism operates via a mechanical spring-loaded trigger system. Upon full actuation, a striker impacts the primer of the selected cartridge, initiating a propellant-driven pressure surge. This force ruptures a burst disk, propelling a liquid stream of oleoresin capsicum (OC) through the nozzle at high velocity. The resulting spray pattern spans approximately 0.3 m in diameter at 5 m distance and up to 0.6 m at the device’s maximum effective range of 7 m. A minimum safe operating distance of 1.5 m is mandatory to prevent severe ocular or dermal injury due to the kinetic energy of the liquid jet.

The active agent, OC (capsaicin extract), induces an immediate and intense physiological reaction upon contact with mucous membranes characterized by temporary blindness, coughing, irritation, and acute discomfort. Each magazine is single-use and must be disposed of after four discharges. The JPX6 may be equipped with optional integrated laser sights or open mechanical sights for targeting precision. Given its high discharge energy and focused spray trajectory, the JPX6 offers an effective yet potentially injurious alternative for close-range defense, necessitating proper training and adherence to safety protocols [4].

## Methods

### JPX6 jet protector manufacturer information

According to the manufacturer’s manual (pages 3–5), the JPX6 Jet Protector is not considered safe for use at distances below 1.5 m. The manufacturer warns that closer-range use may result in significant injury [4].

### Ballistic soap test setup

Ballistic soap blocks provided by Carl Alois Walde GmbH & Co KG were used for testing. Each block measured 25 × 25 × 40 cm and weighed 27 kg. Test shots were fired from defined distances, and both liquid dispersion and the resulting physical damage were photo-documented and analyzed: (Table 1)

**Table 1:**
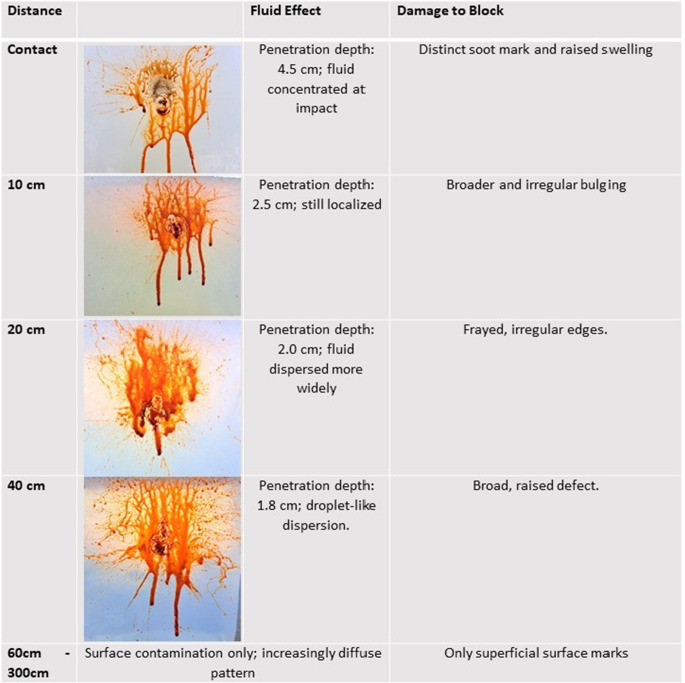
Sequential Documentation of ballistic soap defects at firing distances of 0–40 cm

### Additional observations

The shooter was consistently contaminated by the jet fluid at distances up to 100 cm. The spatial distribution of the liquid allowed for reconstruction of the firing position. Although the fluid contains color pigments, they proved easily washable.

### Analytical methods - shooting distance estimation

Defect areas (~ 2 cm diameter) were sampled using TDX cleanroom swabs and analyzed for chemical residues. The following procedures were employed:

- LC-MS/MS after methanol extraction: detection of Akardit II, DPA, and Capsaicin.
- ICP-MS after nitric acid extraction: detection of Sb, Ba, and Pb.

### Results

- Metallic components were detectable up to 100 cm.
- Stabilizers were only detectable up to 40 cm.
- Capsaicin remained detectable up to 300 cm.

**Fig. 4:**
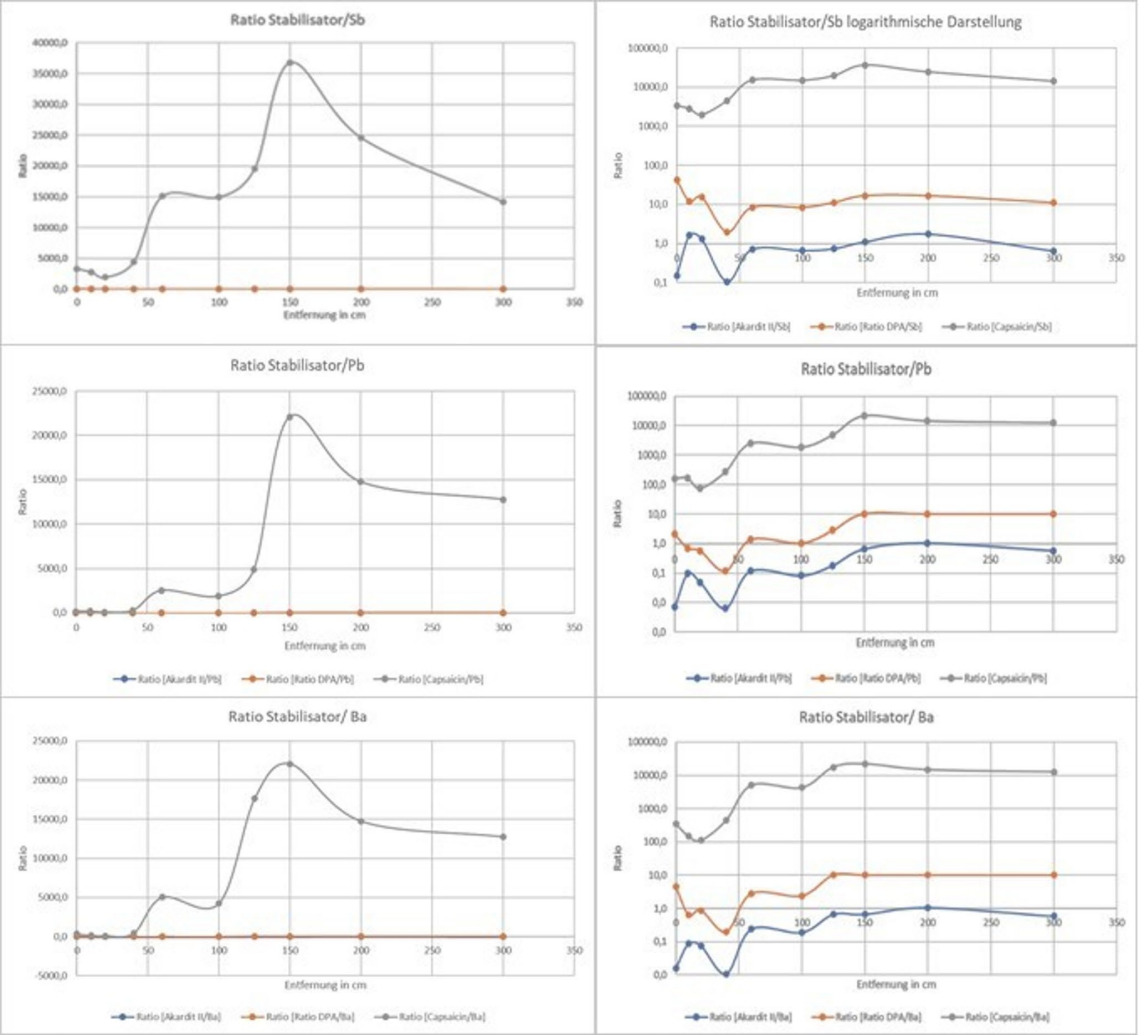
Ratio of stabilizers to metal residues(e.g., DPA/Sb, Akardit II/Ba) and Ratio of capsaicin to metal residues at different distances

This allowed classification into short-range and long-range shots. Using ratio methods (e.g. stabilizer-to-metal, capsaicin-to-metal), accurate distance estimations were possible up to ~ 150 cm; beyond this range, uncertainty increased (Fig. 4).

### Assessment of energy transfer and cavity volume

**Fig. 5:**
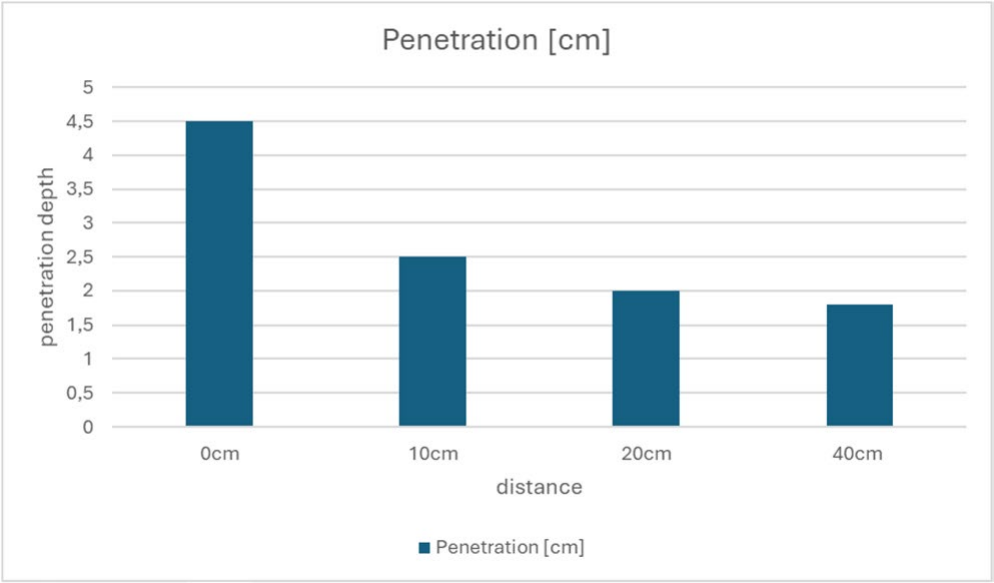
Penetration depth per shot distance (0-40 cm) based on experimental results

The following values describe penetration depth and volumetric defect measurements in ballistic soap: (Table 2, Fig. 5)

**Table 2:**
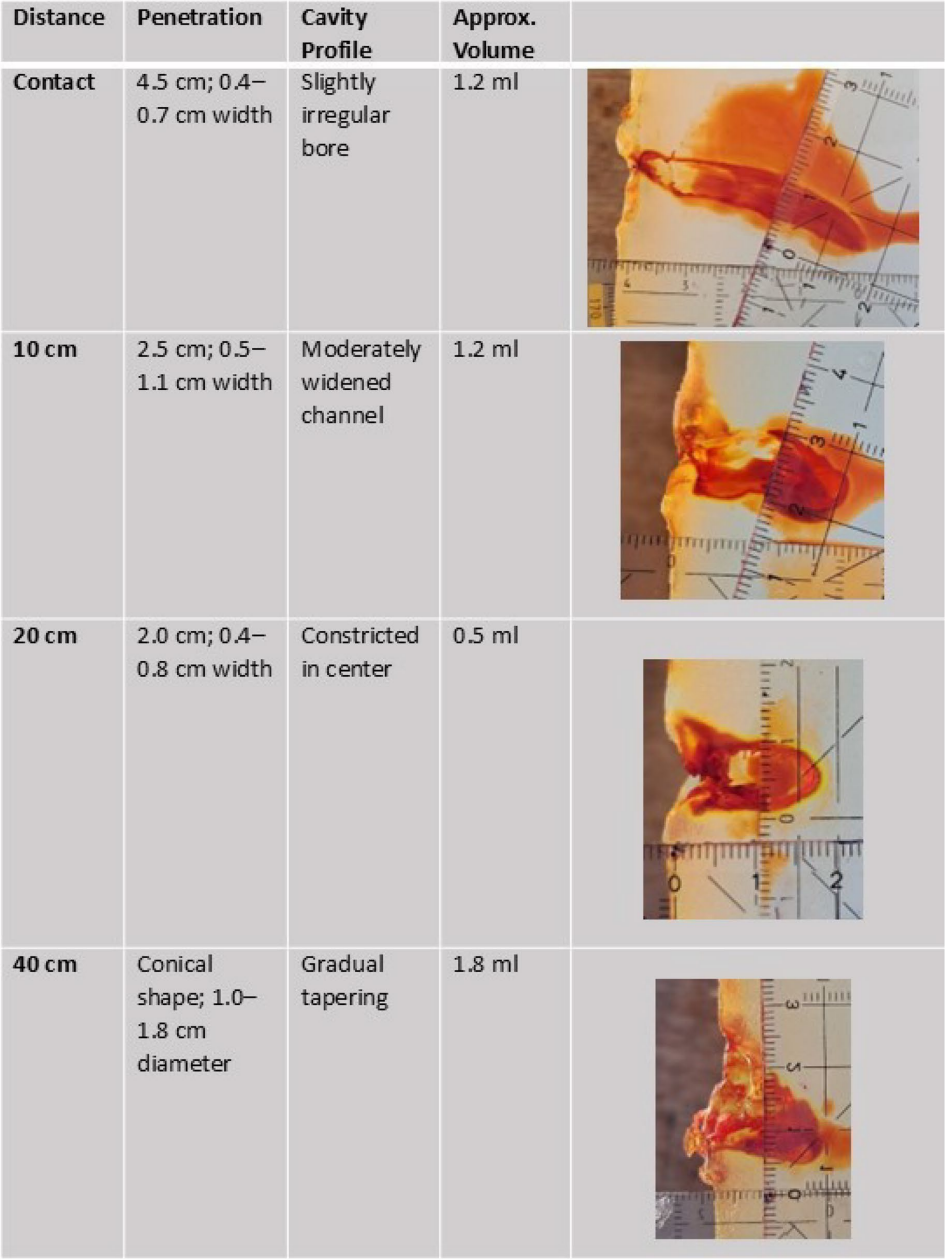
Penetration depth, cavity geometry, and estimated defect volume in ballistic soap blocks at defined shooting distances

## Results and interpretation

Shots fired at distances below the recommended safety range of 1.5 m resulted in measurable penetration of ballistic soap. At point-blank range, the liquid jet reached a depth of 4.5 cm; at 10 cm, 2.5 cm; at 20 cm, 2.0 cm; and at 40 cm, 1.8 cm. These findings clearly demonstrate the capability of the JPX6 device to breach the surface and impact underlying soft tissues at short distances.

### Interpretation

When extrapolated to the anatomy of an average, unclothed adult human body, shots with this energy level pose a significant risk for serious or even fatal injuries:

- Ocular exposure could lead to permanent visual loss or globe rupture. Penetration of the orbital cavity may allow direct contact with brain tissue.
- Cervical shots could reach major blood vessels, risking instant hemorrhage or air embolism.
- Thoracic penetration could compromise lungs or the heart, resulting in tension pneumothorax, hemopericardium, or fatal cardiac injury.
- Abdominal impacts might rupture the liver, spleen, intestines, leading to internal bleeding and death.
- Genital injuries may cause permanent tissue damage and infertility.
- Retroperitoneal impact could lacerate kidneys, producing concealed but life-threatening hemorrhage.
- Inguinal exposure, as observed in our case, poses a high risk due to the superficial course of large-caliber vessels.

Additionally, Piexol 400 kS - containing oleoresin capsicum with a pungency of 400,000 Scoville units - can damage mucous membranes, skin, and eyes. Systemic toxicity is conceivable [8]. Capsaicin also exerts cytotoxic effects on keratinocytes [9], compromising skin integrity. When applied to damaged or penetrated tissue, it may further harm deep structures and promote delayed wound healing or bacterial infection [10].

Shots from ≥ 60 cm caused only superficial damage, but resulted in much wider surface contamination by jet fluid. In confined environments, even the shooter was exposed to irritant backflow at ranges below 1 m.

## Discussion

Pepper spray launchers occupy a complex position within the spectrum of defensive tools. Although categorized as “less-lethal,” our findings and previously reported cases challenge the adequacy of this label in forensic and legal contexts.

The injury mechanisms observed in this case bear striking resemblance to those produced by firearms, both in external morphology and internal damage consistent with injuries caused by weapons in the classical sense. Entry wounds with abrasion collars, thermal and mechanical tissue destruction, and vascular lacerations are hallmarks of high-velocity impact, regardless of whether caused by bullets or high-pressure irritant jets. As such, pepper spray launchers particularly at close range blur the line between less-lethal deterrents and potentially fatal weapons.

The existing literature supports this perspective. In 2022, Weber et al. [6] reported a penetrating injury caused by a JPX launcher, and Borges et al. [7] described a fatality due to high-velocity jet-induced trauma from a less-lethal weapon. These cases reinforce that pepper spray guns must be considered within the forensic differential diagnosis when no projectile is recovered but trauma suggests high-energy directional force.

From a forensic standpoint, our study highlights several critical considerations:

- Crime scene interpretation: The presence of a pepper spray gun at the scene must trigger a complete forensic reconstruction, including trajectory estimation, chemical residue analysis, and mucosal or dermal examination for capsaicinoid contamination.
- Chemical analytics as forensic tools: Our ratio-based analysis of stabilizers and metal residues (LC-MS/MS and ICP-MS) enabled a retrospective estimation of shooting distance. This methodology could help clarify ambiguous cases or confirm witness testimony.
- Legal classification and policy: As these weapons are legally available without specialized training, the risk of accidental or intentional misuse is substantial. Legislators may need to reevaluate the current classification, particularly in light of their documented lethality at close range.
- Medical and forensic documentation: We recommend the use of capsaicinoid screening, swab sampling, and injury pattern recognition protocols in all unexplained superficial lesions with orange discoloration, thermal borders, or mucosal damage.

In conclusion, the JPX6 pepper spray launcher exemplifies the limitations of the “less-lethal” label in modern forensic practice. Comprehensive evaluation integrating physical, chemical, and contextual evidence is essential to accurately determine the role of such weapons in violent or fatal incidents.

## Conclusion

Pepper spray launchers such as the JPX6 are widely available for purchase - often online, at affordable prices - and legally accessible to persons over 18 years of age. In public perception, they are often equated with harmless devices.

However, our findings challenge this assumption. The JPX6 must be considered a potentially lethal weapon when discharged at short range, especially by untrained users or in confined spaces.

We strongly recommend that:

- forensic experts regard such devices as possible homicide weapons,
- any orange discoloration on the skin be swabbed for capsaicinoid or gunshot residue testing, and.
- pepper spray-related injuries be evaluated with the same rigor as firearm injuries - including examination for entry wounds, soot deposition, stretch zones, and chemical traces.

According to our evidence, pepper spray gunshot injuries can mimic those of conventional firearms - and must be treated with corresponding caution.
